# Supplementary material for: Body Composition Analysis in Perimenopausal Women Considering the Influence of Vitamin D, Menstruation, Sociodemographic Factors, and Stimulants Used
Source: Int J Environ Res Public Health. 2022 Nov 28;19(23):15831. doi: 10.3390/ijerph192315831 (PMC9736338; doi:10.3390/ijerph192315831)
Supplement: Supplementary file 1 [file ijerph-19-15831-s001.zip › ijerph-1996242-supplementary.pdf]

International Journal of
Environmental Research and Public Health

MDPI

Suplementary Materials

Table S1. Body composition analysis in relation to place of residence.

Place of residence / Body composition	Country (n=17)		City up to 10 thousand residents (n=6)		City up to 100 thousand residents (n=20)		City above 100 thousnad residents (n=147)		H	*p*
	**Mdn**	**IQR**	**Mdn**	**IQR**	**Mdn**	**IQR**	**Mdn**	**IQR**		
Adipose tissue mass [kg]	34.9	6.8	29.4	7.9	34.5	10.2	33.2	7.5	3.277	0.651
Adipose tissue mass [%]	7	2	5.5	3	7.5	4	7	4	4.111	0.25
Visceral adipose tissue	51	12	37	14	50	23.5	46	18	5.059	0.168
Metabolic age [years]	27.3	6.5	25.2	8.3	28.5	6.5	25.4	6	3.146	0.37
Muscle mass [kg]	43.2	10.4	45.9	7.4	46.9	7.6	44.7	6.5	1.626	0.653
Osseous tissue mass [kg]	2.3	0.6	2.5	0.4	2.5	0.5	2.4	0.4	1.7	0.638
Water content [kg]	46.1	4.6	50.3	5.3	46.6	7.3	47.3	5.3	3.48	0.323
Water content [%]	5.6	0.5	5.5	0.6	5.7	0.5	5.6	0.6	2.1	0.552
Phase angle [º]										

n – numer of respondents; **Mdn** – median; **IQR** – interquartile range; **H** – harmonic mean; **p** – test probability.

Table S2. Body composition analysis in relation to marital status in studied women.

Marital status / Body composition	Formal relationship (n=135)		Informal relationship (n=24)		Single (n=31)		H	*p*
	**Mdn**	**IQR**	**Mdn**	**IQR**	**Mdn**	**IQR**		
Adipose tissue mass [kg]	33.7	8.3	34.1	5.8	32.2	10.2	1.308	0.52
Adipose tissue mass [%]	7	3	7	2.5	7	4	0.632	0.729
Visceral adipose tissue	47.5	19.5	47.5	14	46	13	0.322	0.851
Metabolic age [years]	26.1	5.7	25.4	6.3	24.9	7.4	0.865	0.649
Muscle mass [kg]	45.5	6.6	43.7	7.8	43.9	7.3	0.388	0.824
Osseous tissue mass [kg]	2.4	0.4	2.3	0.4	2.3	0.3	0.855	0.745
Water content [kg]	47	6.1	46.8	3.9	47.8	8.5	0.522	0.771
Water content [%]	5.6	0.6	5.6	0.5	5.6	1.3	1.338	0.512
Phase angle [º]								

n – numer of respondents; **Mdn** – median; **IQR** – interquartile range; **H** – harmonic mean; **p** – test probability**.**

Table S3. Body composition analysis in relation to professional activity in studied women.

Professional activity / Body composition	Active (n=168)		Not active (n=23)		Z	*p*
	**Mdn**	**IQR**	**Mdn**	**IQR**		
Adipose tissue mass [kg]	33.6	6.8	34.4	14.2	-0.543	0.588
Adipose tissue mass [%]	7	2	8	5	-1.182	0.238
Visceral adipose tissue	47	15.5	50	21	-1.776	0.076
Metabolic age [years]	25.7	5.9	26	8.8	-0.036	0.97
Muscle mass [kg]	45.5	6.6	43.2	8.1	1.192	0.235
Osseous tissue mass [kg]	2.4	0.4	2.3	0.4	1.255	0.21
Water content [kg]	47.1	4.9	46.3	10.1	0.593	0.555
Water content [%]	5.6	0.6	5.6	0.6	0.147	0.884
Phase angle [º]						

n – numer of respondents; **Mdn** – median; **IQR** – interquartile range; **Z** – normal distribution; **p** – test probability.

Table S4. Body composition analysis in relation to smoking tobacco in studied women.

Smoking tobacco / Body composition	Yes (n=25)		No (n=166)		Z	*p*
	**Mdn**	**IQR**	**Mdn**	**IQR**		
Adipose tissue mass [kg]	33.1	6.5	33.6	7.7	0.373	0.71
Adipose tissue mass [%]	7	3	7	3	0.301	0.765
Visceral adipose tissue	46	18	48	17	0.019	0.983
Metabolic age [years]	26.5	7.1	25.7	5.7	0.039	0.968
Muscle mass [kg]	44.5	6.4	45.1	6.8	0.091	0.928
Osseous tissue mass [kg]	2.4	0.4	2.4	0.4	-0.072	0.943
Water content [kg]	47.6	4.7	47.1	5.9	-0.262	0.795
Water content [%]	5.6	0.6	5.6	0.6	0.274	0.786
Phase angle [º]						

n – numer of respondents; **Mdn** – median; **IQR** – interquartile range; **Z** – normal distribution; **p** – test probability.
